# Supplementary material for: MetaRibo-Seq measures translation in microbiomes
Source: Nat Commun. 2020 Jun 29;11:3268. doi: 10.1038/s41467-020-17081-z (PMC7324362; doi:10.1038/s41467-020-17081-z)
Supplement: Supplementary file 10 — Supplementary Data 7 [file 41467_2020_17081_MOESM10_ESM.zip › File2/Confidence_High_Taxonomy/246898_out.krona.html]

Javascript must be enabled to view this page.

members
magnitude
magnitudeUnassigned
count
unassigned
taxon
rank

246898\_out

6

2
6
superkingdom

6
1239
phylum

186801
6
class

186802
6
order

4
186803
family

genus
3
572511

species

SRS019068\_contig\_number\_48552SRS050422\_contig\_number\_26744SRS1041091\_contig\_number\_22124
3
1226324

species
1
658086

SRS143991\_contig\_number\_24197

541000
2
family

2
1263
genus


SRS1041136\_contig\_number\_contig-100\_5268.81112
2293228
1
species

2293188
1

SRS077502\_contig\_number\_13054
species
